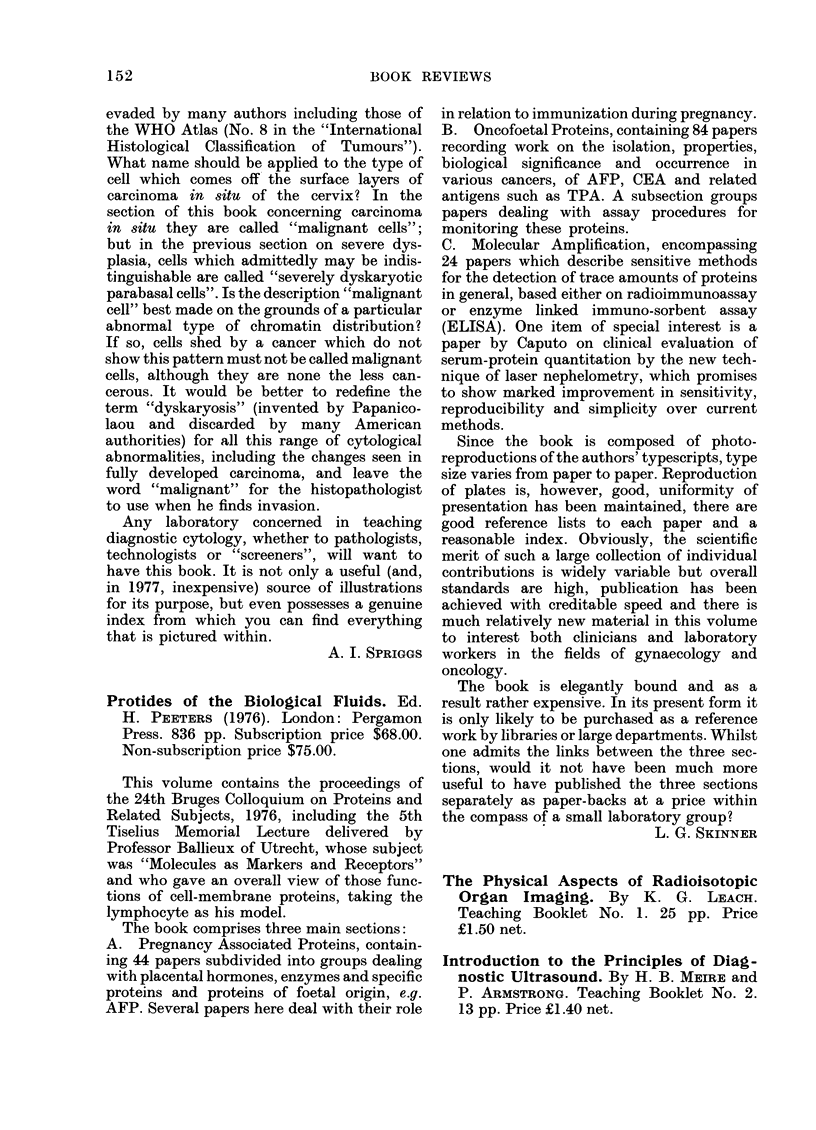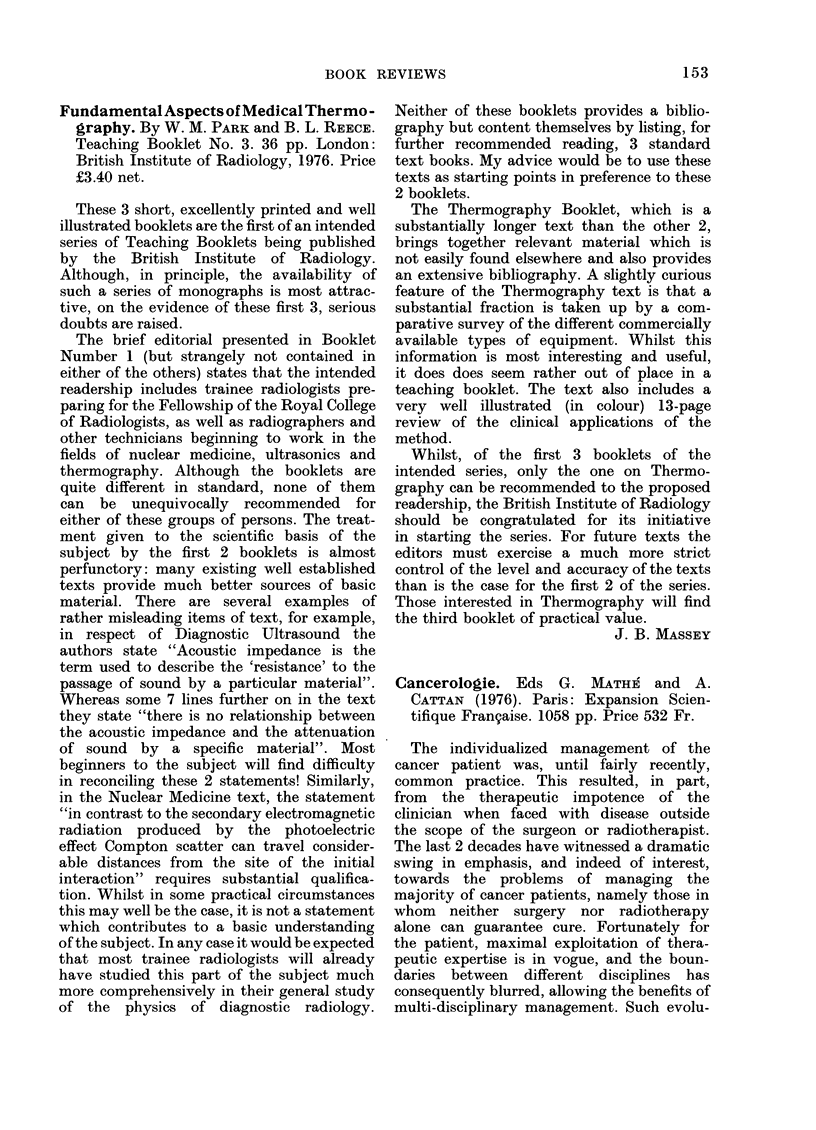# The Physical Aspects of Radioisotopic Organ Imaging

**Published:** 1977-07

**Authors:** J. B. Massey


**The Physical Aspects of Radioisotopic Organ Imaging.** By K. G. Leach. Teaching Booklet No. 1. 25 pp. Price £1.50 net.

**Introduction to the Principles of Diagnostic Ultrasound.** By H. B. Meire and P. Armstrong. Teaching Booklet No. 2. 13 pp. Price £1.40 net.

**Fundamental Aspects of Medical Thermography.** By W. M. Park and B. L. Reece. Teaching Booklet No. 3. 36 pp. London: British Institute of Radiology, 1976. Price £3.40 net.

These 3 short, excellently printed and well illustrated booklets are the first of an intended series of Teaching Booklets being published by the British Institute of Radiology. Although, in principle, the availability of such a series of monographs is most attractive, on the evidence of these first 3, serious doubts are raised.

The brief editorial presented in Booklet Number 1 (but strangely not contained in either of the others) states that the intended readership includes trainee radiologists preparing for the Fellowship of the Royal College of Radiologists, as well as radiographers and other technicians beginning to work in the fields of nuclear medicine, ultrasonics and thermography. Although the booklets are quite different in standard, none of them can be unequivocally recommended for either of these groups of persons. The treatment given to the scientific basis of the subject by the first 2 booklets is almost perfunctory: many existing well established texts provide much better sources of basic material. There are several examples of rather misleading items of text, for example, in respect of Diagnostic Ultrasound the authors state "Acoustic impedance is the term used to describe the 'resistance' to the passage of sound by a particular material". Whereas some 7 lines further on in the text they state "there is no relationship between the acoustic impedance and the attenuation of sound by a specific material". Most beginners to the subject will find difficulty in reconciling these 2 statements! Similarly, in the Nuclear Medicine text, the statement "in contrast to the secondary electromagnetic radiation produced by the photoelectric effect Compton scatter can travel considerable distances from the site of the initial interaction" requires substantial qualification. Whilst in some practical circumstances this may well be the case, it is not a statement which contributes to a basic understanding of the subject. In any case it would be expected that most trainee radiologists will already have studied this part of the subject much more comprehensively in their general study of the physics of diagnostic radiology. Neither of these booklets provides a bibliography but content themselves by listing, for further recommended reading, 3 standard text books. My advice would be to use these texts as starting points in preference to these 2 booklets.

The Thermography Booklet, which is a substantially longer text than the other 2, brings together relevant material which is not easily found elsewhere and also provides an extensive bibliography. A slightly curious feature of the Thermography text is that a substantial fraction is taken up by a comparative survey of the different commercially available types of equipment. Whilst this information is most interesting and useful, it does does seem rather out of place in a teaching booklet. The text also includes a very well illustrated (in colour) 13-page review of the clinical applications of the method.

Whilst, of the first 3 booklets of the intended series, only the one on Thermography can be recommended to the proposed readership, the British Institute of Radiology should be congratulated for its initiative in starting the series. For future texts the editors must exercise a much more strict control of the level and accuracy of the texts than is the case for the first 2 of the series. Those interested in Thermography will find the third booklet of practical value.

J. B. Massey